# Development of a distributed group control strategy for pumping well groups connected by multisource DC microgrids

**DOI:** 10.1038/s41598-024-56018-0

**Published:** 2024-03-05

**Authors:** Jixiang Yue, Zhenhua Sun, Haoguang Li, Wenyu Zhu, Fengming Li, Zhenjie Wang

**Affiliations:** 1 Shandong Institute of Petroleum and Chemical Technology, No 271, Beier Road, Dongying, 257061 Shandong China; 2 Sinopec Shengli Petroleum Management Co., Ltd. New Energy Development Center, Dongying, Shandong 257017 China

**Keywords:** Multisource DC microgrid, Pumping unit group control, Distributed group control strategy, Power weighting, Group weighted moving average, Energy grids and networks, Electrical and electronic engineering, Solar energy

## Abstract

Due to the alternating loads on pumping units and the integration of new energy sources, multisource DC microgrid pumping unit well groups experience increased fluctuations in voltage and power as well as superimposed peak and valley values. This work presents a distributed control strategy for pumping unit well groups on a multisource DC microgrid based on the weighted moving average algorithm. A centralized control program is implanted in the RTU of the single-well controller of each pumping unit, and communication with each well is realized via SCADA and multicast communication, resulting in a distributed well group system. The real-time power values of the pumping well group are calculated by grouping the power values, and each group is weighted using the total power fluctuation threshold of the well group as the control target. Then, a weighted moving average algorithm is used to predict the next power value and form a table of predicted real-time power spectra. According to the power values in the community power spectrum table, the inverter frequency is proportionally adjusted downwards to reach the power peak before deceleration; after the power peak is crossed, the frequency is increased in the same way to reach the power valley before acceleration. Finally, the peak and valley power values of the bus system level off and further learn to reach the set impulse; ultimately, a stable impulse is formed. In laboratory testing and field application in the Shengli Oilfield XIN-11 block, the group control software module effectively suppressed the active power peak and valley values and voltage fluctuations of the bus system, the active power fluctuation rate range decreased by more than 70%, and the DC bus voltage fluctuation range decreased by more than 80%; moreover, the active power decreased by approximately 6% without additional hardware costs.

## Introduction

For future intelligent power distribution systems, DC microgrid technology is being developed; this approach is important for promoting energy conservation, emission reduction and sustainable energy development^1–3^. Oilfield production systems, as major energy consumers, have been actively implementing this new energy strategy. Based on the oilfield supervisory control and data acquisition (SCADA) system, multisource DC microgrids for oilfield production have been constructed^4–7^. Multisource DC microgrids for well groups in oil production have been used to distribute energy from newer types of energy sources such as wind power and photovoltaic power^8–11^. We formed a well group with multiple pumping units at similar distances and with some common attributes. DC bus power supply components were used to incorporate several new energy sources such as wind power and photovoltaic power to form a small distributed multisource DC microgrid for the well group. The DC microgrid connects the control cabinets of multiple pumping units in parallel through the DC buses, and the integrated inverter and remote terminal unit (RTU) in each control cabinet can realize mutual feed sharing and recycling of the power generated by multiple pumping units^12–15^. The pumping units exhibit commonalities and mutually influence each other^16–19^. Because the pumping units are under alternating load, peaks and valleys are superposed, causing various hazards and necessitating a unified control and management system. This paper presents a proposal for a distributed group control strategy for a block oil recovery multisource DC microgrid and describes the onsite implementation of the method to test its effectiveness.

## Multisource DC microgrid group control research status and current problems

### Existing problem

The use of well group DC microgrids in oil production systems has expanded rapidly in recent years. By the end of 2020, more than 2000 oil wells in the Shengli Oilfield had transitioned to DC bus power sources, which exhibit outstanding technological and financial advantages. However, the overall aim for this "group without control" is to obtain the right feedback and a fair amount of the power generated by each pumping unit. Achieving "group control" has a long way to go before these objectives are met^8–10^.

Due to the alternating load characteristics of pumping units, reverse power generation often occurs due to phase reversal, and the superposition of peaks and valleys occurs when the DC buses of pumping units operate as a group. Significant voltage and active power fluctuations occur, and the peak-to-valley ratio of the DC bus power increases. Thus, a DC microgrid system might result in feedback overvoltage, undervoltage, and overload. This makes the integration of wind energy, photovoltaics, and other emerging energy sources into the DC bus microgrid system difficult. Harmonics have been a serious concern with the introduction of these new energy sources, as they can cause the system to shut down or cause explosions in capacitors, insulated gate bipolar transistors (IGBTs), and other powerful electronic equipment, resulting in hazardous events and decreases in oil well productivity^9,10^. Therefore, unified control and management of the operation of DC bus pumping units in oil wells are urgently needed. This approach can help coordinate the operation status of each pumping unit, suppressing the total peak and valley values of the bus and achieving stable and safe system operation.

### Main research progress

In recent years, the DC microgrid group control strategy has been a popular research topic. Zare and Derbas et al. improved the hybrid artificial bee colony algorithm and differential evolution algorithm, as optimization technologies, to address the problem of optimal energy management for grid-connected microgrids^20,21^. Kim et al.^22^ proposed a DC series arc detection algorithm in a photovoltaic (PV) system using an adaptive moving average (AMA), which is effective for centralized and spread-type frequency fluctuations. Dong et al.^23^ proposed a combined model, i.e., a wind power prediction model based on a multiclass autoregressive moving average (ARMA), that has a two-layer structure of wind power data with a logistic function-based classification method and a prediction algorithm in each class. Wang et al.^24^ proposed a distributed Kalman filter power prediction algorithm combined with graph theory. Nguyen and Kim^25^ proposed a hybrid control method based on distributed consensus control and central model predictive control that could reduce the computational burden of central control methods and the complexity of the communication network in decentralized control strategies. Cheng et al.^26^ designed a modified distributed Kalman filter (KF) based on the mixed phasor measurement unit (PMU) and RTU measurement model that independently estimated local states via local measurements. Munsi et al.^27^ proposed the use of an algorithm based on the unscented Kalman filter (UKF) to control the voltage and current of a microgrid against unknown noise. Che and Shahidehpour^28^ investigated the power flow between a DC microgrid and a main grid from the perspectives of economy, optimization and stability and designed a three-level control strategy for a DC microgrid. However, most of these studies focused on power generation side control rather than DC microgrid internal control, and different user group control objectives require different implementation schemes. At present, no specific system is applied to well group production systems^4,9,19^. Su and Jesse Thornburg et al. selected the total power output variance of a well group as a control target function and used the improved adaptive genetic algorithm to calculate the staggered operating time interval of each pumping unit to ensure that the number of pumping units running in the proper power state and the pumping units running in the reverse power state in the well group were relatively consistent^10,15^. To a certain extent, it is beneficial but unacceptable to change the pumping such that flushing times are the same.

## Distributed power group-weighted predictive group control algorithm

### Application environment

**Figure 1:**
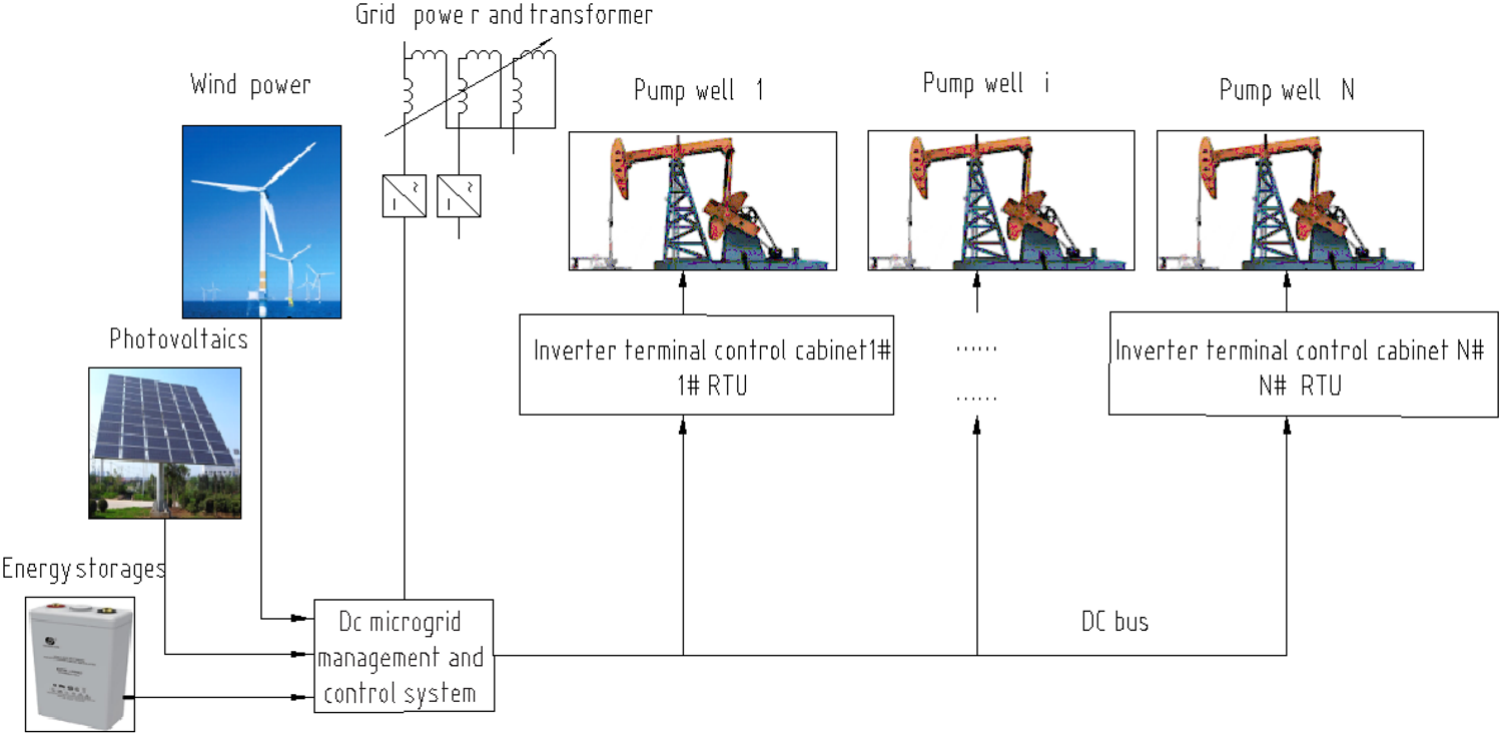
Multisource DC microgrid pumping unit group control system scheme.

The new block oil extraction energy system connects power sources such as grid power, photovoltaic power, wind power, and energy storage to multiple pumping unit loads through a DC microgrid, where RTUs control each well^29–31^. Figure 1 shows the representative control structure of the multisource DC microgrid pumping unit group control system scheme based on SCADA. A control cabinet composed of an inverter module and an RTU is installed in each single well to convert DC into alternating current with variable frequency to realize automatic regulation of the pumping unit motor. The working area of the DC microgrid is generally less than 5 km.

**Figure 2:**
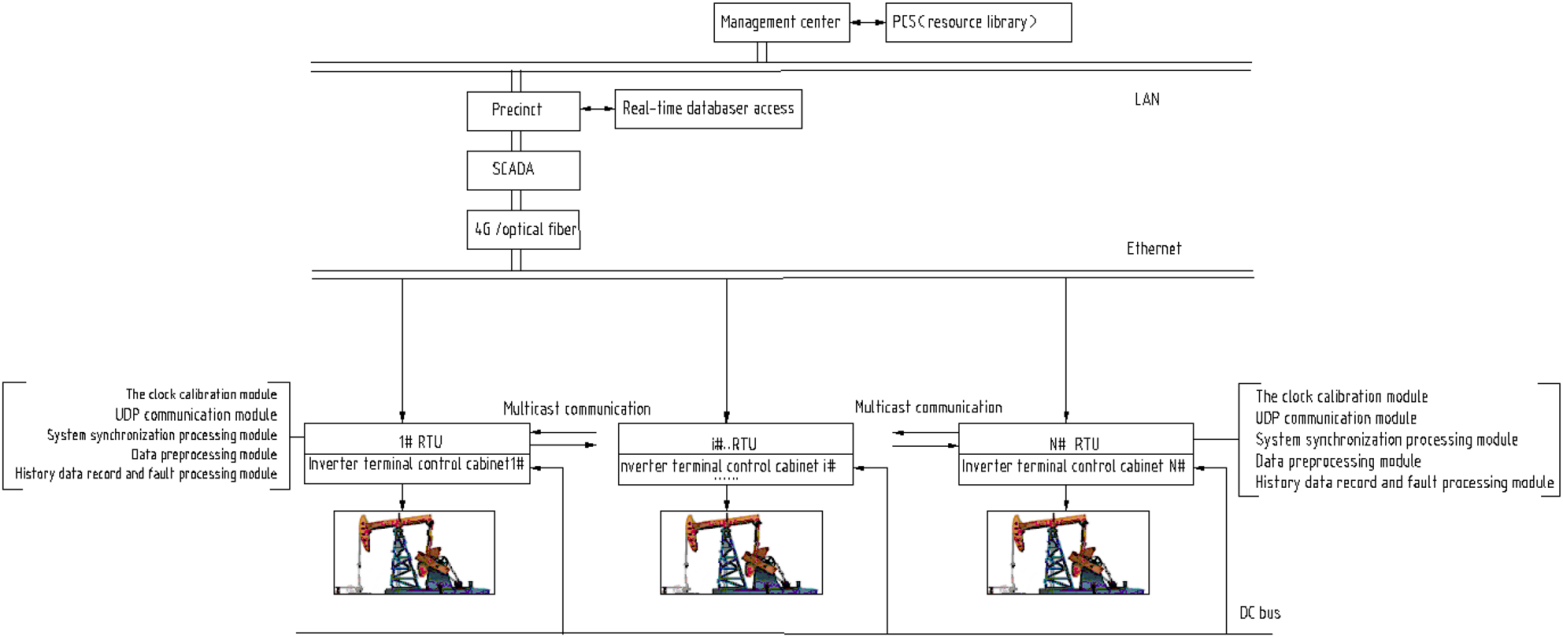
Schematic diagram of the multisource DC microgrid pumping unit group control system after the centralized control elements are implanted in the RTUs.

Figure 2 shows a schematic diagram of the multisource DC microgrid pumping unit group control system after centralized control components are implanted in each RTU. First, centralized control software (hereinafter referred to as "group control software") is implanted into the RTU of each single-well controller of the pumping unit. The RTU includes a central processing unit and memory, without the need for additional hardware. The group control system establishes external communication through the SCADA system, and each well in the well group communicates with the others through multiple cycles, reporting real-time production information about its status, including real-time power, torque, speed, angular acceleration and other production information; each well also receives production information from the other wells. The RTU is connected to the inverter and frequency converter that control the pumping unit motor, controlling the working conditions, thereby constructing a distributed well group control system.

### Group control principle

Because the power of each individual pumping unit within the well group exhibits periodic fluctuations and the power extremes are deterministic, the real-time power values of the pumping unit well group are calculated by grouping the power values and weighting each group. The DC microgrid in question is a distributed system, and the total power fluctuation threshold for the well group is employed as the control objective. In addition, the weighted moving average algorithm is used to predict the next power value to construct a predicted real-time power spectrum table in order to determine the highest weighted pumps, that is, those with the greatest impact on power. According to the power spectrum table of the group, the inverter frequency is proportionally reduced to control the motor to reach the power peak before deceleration. After the power peak is crossed, the frequency is increased in the same way reach the power valley before acceleration. Finally, the power peak and valley values of the bus system level off, further learning to reach the set impulse occurs, and finally, a stable impulse is formed, effectively coordinating the operating states among the pumps^32^. The system is designed to achieve smooth and safe operation.

### Group control algorithm and implementation process

The group control software realizes well group control from within the RTU of each pumping unit controller in the well group. Considering that the real-time pumping power generally fluctuates cyclically, multiple pumping power divisions within a certain interval and a group weighted moving average are used for the prediction analysis (refer to Fig. 5).

An informational connection is created between each well node in the well group, after which the system periodically corrects the time. The system clock calibration and group control error are closely related.

The user datagram protocol (UDP) software sends the RTUs information after power-on, including grouping, clock, distribution of hashtag, IP, marking clock, and member list information. The UDP further increases or decreases the number of members. Management rights are automatically transferred. The address sizes in the network are sorted.

The RTU is connected to the inverter. First, the stored data area code is extracted. Second, the data in each area code are extracted. Finally, the data in the inverter, including real-time power, voltage, current, and impulse, are extracted and stored in the form of a database file. After the well numbers are obtained, information regarding any RTU that is not powered on is sent to the group, and the members of the group are retrieved. Members of the group who are absent for an extended period will receive separate notifications, and the database will create and store the group table file group.jq.

The real-time power spectrum (power.dt) of each pumping unit is determined, and the power weight is subsequently calculated to determine the contribution of the different single-well models to the power of the bus. The RTU data are reread at certain intervals to recalculate the real-time power spectrum of each pumping unit. The weighted calculation formula of the real-time power value is as follows:

1 $$ Q_{j} = \frac{{\sum\nolimits_{{{\text{i}} = 1}}^{{{\text{n}}_{{1}} }} {P_{1j} \times k_{1} ) + \sum\nolimits_{{{\text{i}} = 1}}^{{{\text{n}}_{2} }} {(P_{2j} \times k_{2} ) + \cdots + \sum\nolimits_{i = 1}^{{{\text{n}}_{{\text{n}}} }} {(P_{ij} \times k_{n} )} } } }}{{\sum\limits_{i}^{n} {k_{i} } }} $$

where $Q_{j}$ is the actual real-time power value of a single well and is reported after weighting;

$k_{i}$, grouping weight, $k_{i} { = }n_{i} /N,N = \sum\nolimits_{i = 1}^{j} {n_{i} }$; *i*, group number; $n_{i}$, number of pumping units/group number; $P_{ij}$ is real-time power value of pumping unit i at time j;

$$\vdots $$

$P_{1j} = \left\{ {P_{1j} :min \le Value < S1,n_{1} ,k_{1} } \right\}$, $P_{1j}$, Real-time power value,$n_{1}$, number of $P_{1j}$;

$P_{2j} = \left\{ {P_{2j} :S1 \le Value < S2,n_{2} ,k_{2} } \right\}$, $P_{2j}$, Real-time power value,$n_{2}$, number of $P_{2j}$;

$P_{3j} = \left\{ {P_{3j} :S2 \le Value \le S3,n_{3} ,k_{3} } \right\}$, $P_{3j}$, Real-time power value,$n_{3}$, number of $P_{3j}$;

$$\vdots $$

$P_{ij} = \left\{ {P_{ij} :S(n - 1) \le Value \le Sn,n_{n} ,k_{n} } \right\}$, $P_{ij}$, Real-time power value,$n_{i}$, number of $P_{ij}$;

A real-time community power spectrum table is established for a group of wells and used to make predictions with a group weighted moving average based power prediction model.

2 $$ Q_{j + 1} = \frac{{\sum\nolimits_{{{\text{i}} = 1}}^{{{\text{n}}_{{1}} }} {(P_{1j + 1} \times k_{1} ) + \sum\nolimits_{{{\text{i}} = 1}}^{{{\text{n}}_{2} }} {(P_{2j + 1} \times k_{2} ) + \cdots + \sum\nolimits_{{{\text{i}} = 1}}^{{{\text{n}}_{{\text{m}}} }} {(P_{ij + 1} \times k_{n} )} } } }}{{\sum\limits_{i}^{n} {k_{i} } }} $$

3 $$ \left( {Q_{j + 1} - Q_{j} } \right)/Q_{j} \le A $$

where $A$ is the prediction error threshold, set at 5% or less than 5%.

The inverter is adjusted to level out the power peaks and valleys of the bus system according to the predicted values.

Relevant data, including torque and speed, rotational inertia, angular acceleration, predictions, and postaction prognosis, are updated.

Initial data are input for training; after the model learns for a set number of impulses, a stable impulse table is formed, and continuous operation occurs.

## Implementation

### Hardware support

Without the need for additional hardware resources, the RTU hardware executes the group control software. An ARM11 embedded CPU processor powers the RTU. A software development kit (SDK) for LINUX embedded systems is available on ARM11 devices. The specific development software used is a Windows 10 system and a Linux kernel 3.15. The ARM11 platform supports the TCP/UDP/IP and MODBUSRTU protocols and has RS485, ZIGBEE, and ETHERNET communication interfaces. The device address, connection parameters, and well group data can all be changed online with the ARM11.

### Overall software structure and module interface design

As shown in Fig. 2, the UDP communication module, inverter control module, clock calibration module, data preprocessing module, history data record and fault processing module, well group parameters of the stored file system module, and system synchronization processing module constitute the seven functional modules of the group control software. The RTU multicast communication enables barrier-free information transfer between single wells. The system synchronization processing module realizes synchronization with the oilfield SCADA system, and the data preprocessing module achieves weighting, denoising, and interpolation after frame loss of power values under various pumping units. The group control software adopts an innovative design.

### Laboratory tests

**Figure 3:**
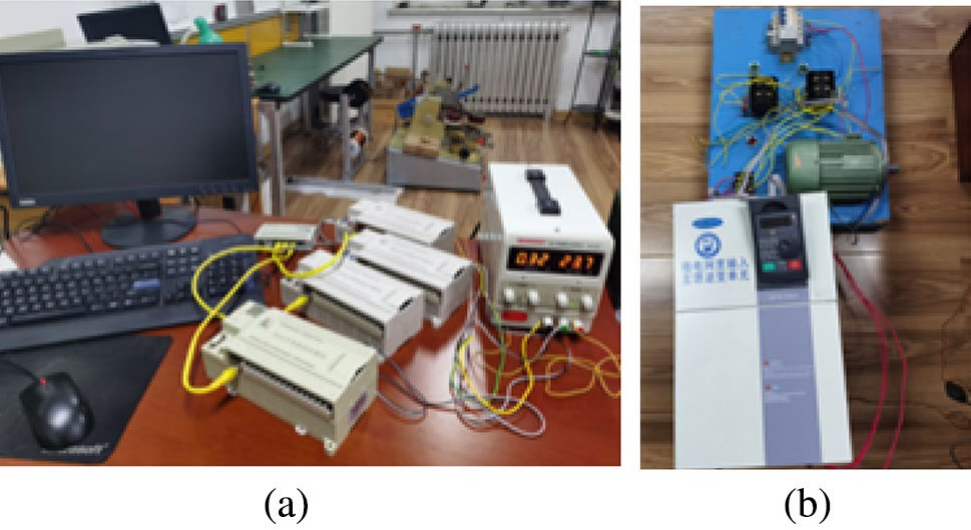
Laboratory test site. (**a**) Panorama of the experimental platform; (**b**) RTU and AC electric motors with a centralized control element implanted**.**

As shown in Fig. 3, an integrated development platform and test platform were set up in the laboratory; these included desktop computers, a raspberry PI 4B, 4 250 W AC electric motors, 4 intelligent monitoring modules for oil and water wells in oilfields of the RTU well group, 4 inverters and a DC power supply. The functionality and dependability of the RS485 communication program, as well as the frequency selection, were tested in a standalone communication environment. The calibration program test was as follows: Run constantly for 10 days, monitor each RTU's time, and ensure that the time inaccuracy is within 0.3 ms. Test the multicast communication on each machine that sends and receives data. The power trend prediction was performed as follows: Predict the power trend in the following minute with less than 5% variance. The bus total power test was as follows: When the automatic control software is implemented, determine the decrease in the total power fluctuations of the buses, where a decrease of 85% in comparison to that of the original system is desirable.

### Field application test

**Figure 4:**
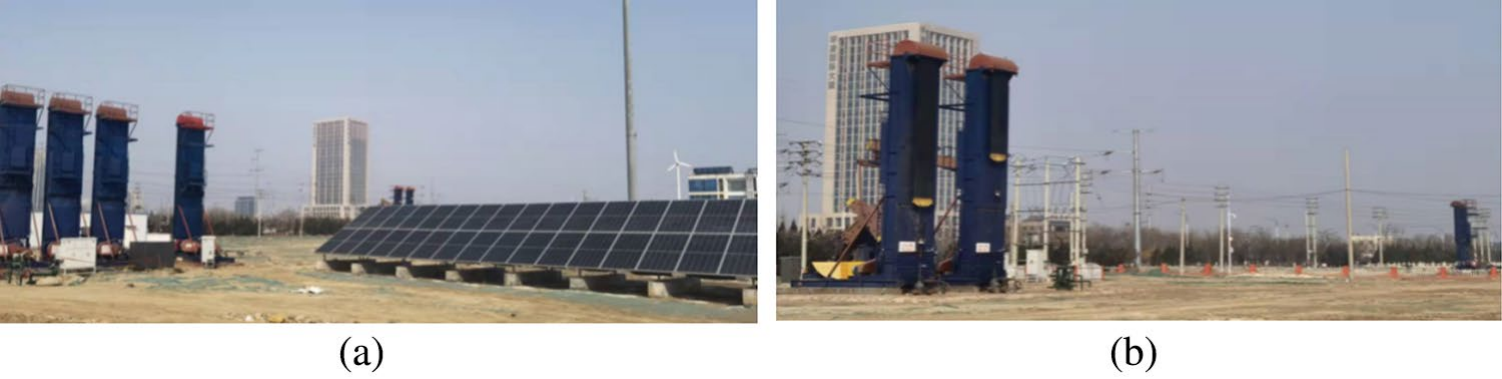
Test site of the DXX11 well group. (**a**) Partial panorama of the well site; (**b**) Participants in testing 4 wells**.**

A portion of the test well site is shown in Fig. 4. The Shengli Oilfield's DXX11 well group, which has a relatively high concentration of 29 wells, was chosen as one of the test locations for the DC micro power grid group control test. According to the relative position, the wells are separated into three groups that are fairly concentrated: well group 1 has four wells, well group 2 has eleven wells, and well group 3 has 14 wells. After the transformer, the grid power is the main power supply, the maximum photovoltaic power is 50 × 2 kW, and the lithium energy storage is 70 kW. Since the photovoltaic power generation at full load is not enough to maintain the power supply of the system, it is used as the supplementary power supply. Well groups 1 and 2 are connected to 50 kW of solar energy, while well group 3 is connected to 50 kW of solar energy. Well group 1 was selected for experimental analysis. Table 1 shows detailed information on the 4 wells.

**Table 1:** Parameter list of pumping units in the DXX11 well group.

	Well number	Motor	Pumping units	P (kW)	Daily power consumption (kW h)	Flush times (min)	IP
1	11XN9	Y225M-8	belt	30	90.17	2.3	192.168.97.124
2	11X173	Y225M-8	belt	30	69.42	3.5	192.168.97.126
3	11X161	YE3-250M-8	belt	37	178.67	2.2	192.168.97.129
4	11X195	Y225M-8	belt	37	192.25	2.1	192.168.97.128

**Figure 5:**
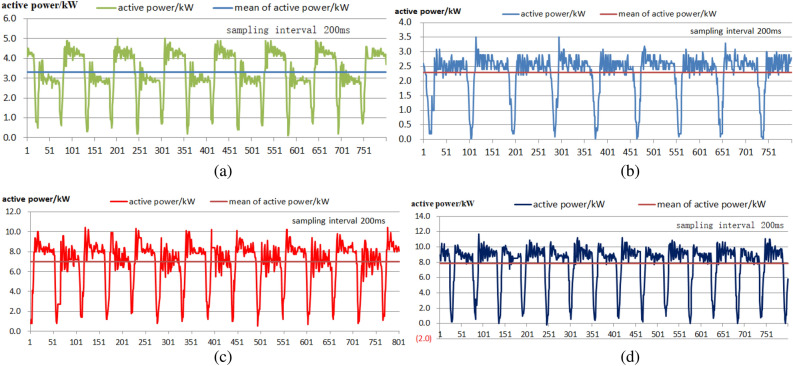
Active power diagram of 4 pumping units after installation of group control software: (**a**)–(**d**) Active power diagrams of pumping units No. 1–4.

Figure 5 shows the actual operation of the four pumping units, and it is obvious that the power of each pumping unit fluctuates significantly, especially for units No. 2 and No. 4, which have negative power values, indicating the existence of "reverse power generation" conditions.

**Figure 6:**
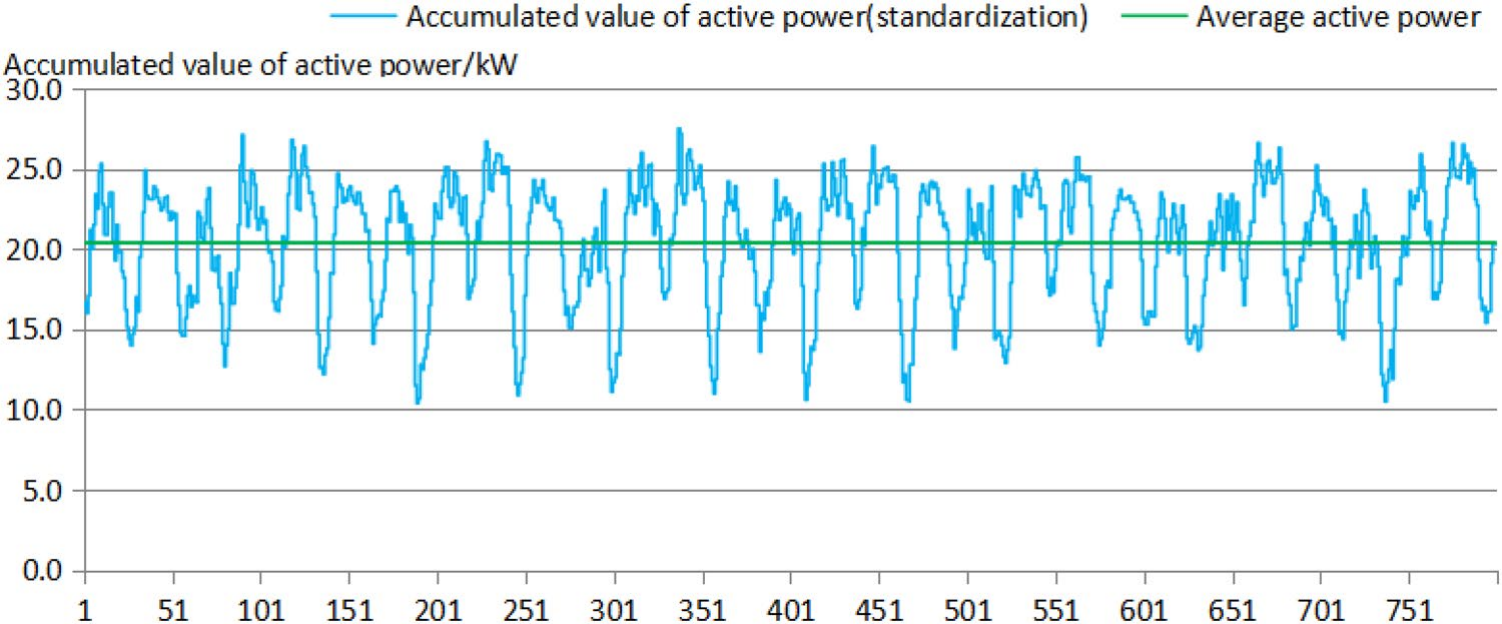
Active power diagram of the test bus after installation of the group control program.

The SCADA system of the Shengli Oilfield was used to test the active power and collect the parameters of each individual well (10:00 a.m., on October 23, 2021). The test sampling interval was 200 ms, with a total of 800 groups of test data and a total length of 160 s. As shown in Fig. 5, the active power of the four pumping units fluctuated from 0.1 to 5.0, 0.0 to 3.5, 0.5 to 10.4 and − 0.2 to 10.7 kW. The active power of each well fluctuated greatly. The mean square errors of the active power were 1.09, 0.73, 2.26 and 2.7 kW. The fluctuation in active power mainly lay in the sharp reduction in active power during the reversing process, and some of these fluctuations even exhibited the "reverse power generation phenomenon". The group control software could not change the original power characteristics of the pumping units. As shown in Fig. 6, the cumulative DC busbar active power of the 4 pumping units fluctuated in the range of 10.4–27.5 kW, with a mean active power of 20.38 kW and a mean variance in the active power of 3.76 kW. The fluctuation range of the active power of the busbars of the four pumping units clearly decreased. The overall strokes of the four pumping units remained the same, but different strokes corresponded to different cycles, such as those in Unit 1. Moreover, periodic fluctuations occurred, mainly due to programmed adjustment of the peak power. In addition, for pumping unit No. 1, there were problems with the balance adjustment of the upstroke and downstroke, and the power difference was quite large.

**Figure 7:**
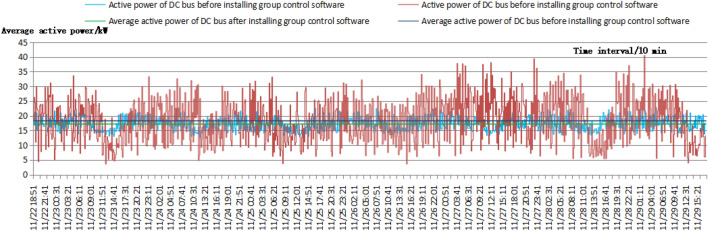
Comparison diagram of the active power fluctuations of the DC bus before and after the installation of the group control software.

**Figure 8:**
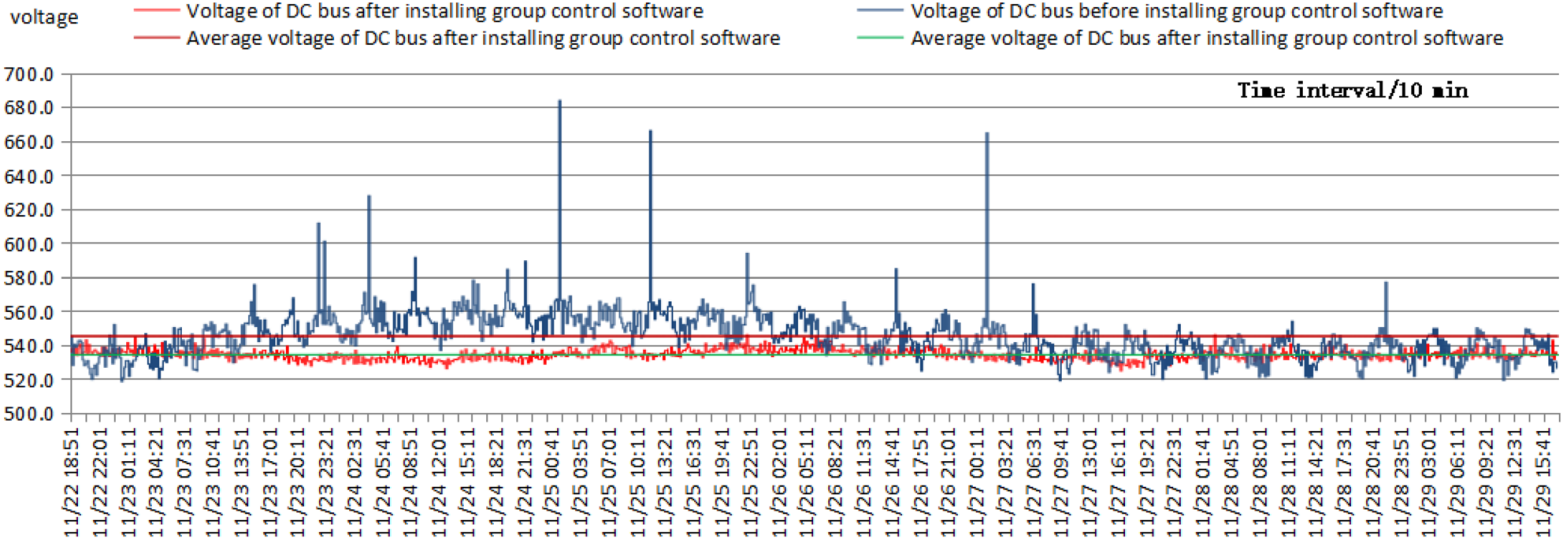
Comparison diagram of the voltage fluctuations of the DC bus before and after the installation of the group control software.

The one-week test was carried out from November 21 to November 28, 2021, to more effectively test the active power and voltage fluctuations of the DC buses. The sampling interval was 10 min, and the sampling time was 167 T. A total of 1000 different datasets was collected. The comparative data consisted of the active power and voltage values of the bridge rectifier power supply output measured prior to installation, where the test voltage was the bus voltage. The weather was sunny during the test, with little variation in lighting. Figure 7 shows that the average active power of the DC bus decreased from a baseline of 18.30–17.22 kW after the group control software was installed, a decrease of 5.90% or an energy savings of 5.90%. The fluctuation range of the active power of the DC bus narrowed from a range of 3.6–40.5 kW to a range of 12.4–23.1 kW, for a 71.01% reduction in the fluctuation range. Figure 8 shows that with the installation of the group control software, the fluctuation range of the DC bus voltage decreased from 684.0–518.6 to 546.1–524.8 V, a decrease of 87.12%, and the mean square deviation of the fluctuation decreased by 77.93%, from 14.09 to 3.11 V. Moreover, the average voltage of the DC bus decreased significantly, from 545.28 to 534.28 V. In particular, significant voltage singularities were removed, which had a substantial impact on the DC microgrid, as the DC bus voltage in the singularity region was much higher than the input voltage of new energy, which had a significant negative impact on the integration of new energy into the DC microgrid; that is, new energy sources such as photovoltaics could not be integrated into the DC microgrid.

## Result analysis

The analysis of the above data shows that the installation of group control software effectively reduces the active power range by more than 70% and the DC bus voltage fluctuation range by more than 80%. It clearly shows that the group control software module levels out the power peaks and valleys of the bus system and decreases the active power fluctuation range.

Additionally, the incorporated DC bus photovoltaic power generation increases, and the DC bus active power decreases by approximately 6% after rectification, with obvious energy savings. The reason for this difference is as follows: due to the use of bridge rectifiers and braking resistors when connected to the grid, the braking resistor consumes some electrical energy when the specified voltage is exceeded. After the group control software is installed, the voltage fluctuation is small, especially after the removal of singular voltage high points, and the braking resistor does not consume electricity.

Analysis of the test data of 11 wells in Group 2 shows that, compared with Group 1, the fluctuation range of the active power of the DC bus decreased by 78.80%, the voltage fluctuation range of the DC bus decreased by nearly 88.92%, and the mean value of the active power of the bus decreased by 6.06%.

## Conclusion

Peak valley superposition occurs in multisource DC microgrids due to the integration of multiple new energy sources and the inherent alternating load characteristics of the pumping units. This phenomenon causes significant fluctuations in the microgrid's bus voltage and active power, which have a negative impact on both the microgrid operation and the influx of multisource new energy. These issues must be addressed.

This article proposes a distributed collaboration strategy based on the group weighted moving average algorithm, which assigns weights to each group and takes the total power fluctuation threshold of the well group as the control objective to form a predicted real-time power spectrum. By regulating the inverter, the motor is controlled to achieve deceleration before the power peak and acceleration compensation after the power peak is crossed, effectively suppressing the power peaks and valleys of the DC bus and further learning the stable set pulses that will arrive later. To confirm that the distributed collaboration strategy was sensible, laboratory testing and on-site application were carried out, utilizing the group weighted moving average algorithm as the foundation for the development of a group control software module.

One significant benefit of this study is that the centralized control software uses the system's built-in RTU implantation to control the pumping well group without requiring additional hardware. This project has significant advantages in optimizing the management of multisource microgrids, increasing the proportion of oilfield green energy and energy savings.

### Supplementary Information

Supplementary Information.

## Data Availability

All data from field testing have been collected and collated to ensure their authenticity and reliability. The datasets used and/or analysed during the current study are available from the corresponding author upon reasonable request. All the data generated or analysed during this study are included in this published article [and its supplementary information files]. For additional clarity, the project team's data collection efforts greatly outstripped the information included in the "Paper field test data and data analysis" submission. To create a remote real-time data collection and monitoring system for the pumping well groups, the project team built upon the supervisory control and data acquisition (SCADA) system. The data were gathered and saved in the cloud by putting remote terminal units (RTUs) in each individual well, photovoltaic substation, maximum power point tracking (MPPT) controller, rectifier power source, etc.
